# Assessment of the Cultural Nuances in COVID-19 Vaccine Uptake Through a Comparative Analysis of English and Spanish Facebook Posts in Tarrant County, Texas: Longitudinal Study

**DOI:** 10.2196/72465

**Published:** 2026-04-27

**Authors:** Ana Aleksandric, Anisha Dangal, Shirin Nilizadeh, Gabriela Mustata Wilson

**Affiliations:** 1 Department of Electrical Engineering and Computer Science, Florida Atlantic University, Boca Raton, FL, United States; 2 Department of Computer Science and Engineering, The University of Texas at Arlington, Arlington, TX, United States; 3 Department of Kinesiology, The University of Texas at Arlington, 701 S Nedderman Dr, Arlington, TX, United States; 4 Louisiana Center for Health Innovation, University of Louisiana at Lafayette, 635 Cajundome Blvd., Lafayette, LA, 70506, United States, 1 337-482-0197

**Keywords:** social media, COVID-19 vaccine hesitancy, cultural nuances, Hispanic population, misinformation, public health interventions, vaccination acceptance

## Abstract

**Background:**

Prior studies have identified key factors contributing to COVID-19 vaccine hesitancy, including concerns over vaccine safety, potential side effects, and mistrust in the health care system. According to the World Health Organization, vaccine hesitancy is among the top 10 threats to global public health. Previous research has suggested that vaccine hesitancy is a significant barrier within the Hispanic population, particularly in Texas.

**Objective:**

This longitudinal study examined the relationships of daily stance, misinformation, and topics in vaccine-related English and Spanish Facebook posts with daily vaccination rates in Tarrant County, Texas, during 2021 and 2022. The goal was to identify the predictors associated with vaccination uptake and inform targeted social media interventions, with particular attention to the Hispanic population.

**Methods:**

COVID-19 vaccine-related English and Spanish Facebook posts from Tarrant County were collected for 2021 and 2022. The study analyzed 12,395 English posts and 1123 Spanish posts. Posts were annotated using GPT-4 for stance, misinformation, and relevant topics, including vaccine availability, safety, and side effects. Category prevalence was compared across English and Spanish posts and across years. Linear regression models were used to examine associations between post characteristics and daily vaccination rates in the total and Hispanic populations.

**Results:**

Regression analysis identified distinct predictors of Hispanic vaccination uptake, including encouraging posts (*P*=.02) and religion-related posts (*P*=.007), which were not significant predictors for vaccination uptake in the general population. A substantial proportion of Spanish discouraging posts focused on vaccine side effects (13/70, 19%) and health system distrust (24/70, 34%), suggesting concerns that may be especially relevant within the Hispanic community. Predictors associated with higher uptake in both the Hispanic and total populations included posts related to vaccine availability (*P*=.01), vaccine safety (*P*=.006), and misinformation debunking (*P*<.001).

**Conclusions:**

Posts related to vaccine availability, vaccine safety, and debunking misinformation were associated with higher vaccination uptake. Encouraging posts and religion-related posts were associated with higher vaccination uptake in the Hispanic population, suggesting meaningful cultural nuances. These findings support the value of culturally tailored social media messaging in public health campaigns.

## Introduction

### Background

In March 2020, the World Health Organization (WHO) declared COVID-19 a pandemic, underscoring the critical need for effective public health measures, including vaccination, to control the spread of the virus [1]. Vaccination has historically been one of the most effective interventions in preventing infectious diseases and protecting population health [2]. Despite the availability and widespread administration of COVID-19 vaccines, many individuals remain hesitant to receive them, raising serious public health concerns [3]. WHO identified vaccine hesitancy as one of the top 10 global health threats in 2019, even before the onset of COVID-19 [4]. This persistent hesitancy has been driven by factors, including vaccine safety concerns, fears of side effects [1], mistrust in the health care system, various socioeconomic and demographic influences [5,6], and the usage of social media platforms for gathering information [7,8].

These factors do not affect all communities equally. Prior research has shown higher vaccine hesitancy in some ethnic minority populations [9]. Studies examining racial and ethnic disparities in COVID-19 vaccination found that people with lower income or education, women, and some minority groups, including Black and Hispanic populations, were more likely to delay or avoid vaccination [10,11]. Such findings underscore the importance of localized analysis, since state- and national-level patterns may mask community-specific concerns.

This is particularly relevant in Texas. During the 2021 legislative session, vaccine hesitancy discussions reflected concerns about medical freedom, vaccine effectiveness, and vaccine safety [12]. While these concerns overlap with national patterns, they may not fully capture barriers affecting smaller communities, including Hispanic populations. National-level data have shown that Hispanic individuals were less likely than White individuals to receive an initial vaccine dose [13]. Other research has suggested that messaging emphasizing family protection and directly addressing mistrust may be more effective for Latino communities [14]. Together, these findings suggest that interventions may need to be tailored to not only demographic groups but also local settings.

Social media has been widely used to study COVID-19 vaccine hesitancy [15-18]. Some studies focused on sentiment [19-25], whereas others examined misinformation [16,26,27]. Prior work has analyzed English-only [28,29], Spanish-only [30-33], and multilingual datasets [34-37]. For example, one study examined vaccine misinformation in English and Spanish [34], and another showed that multilingual discussion around AstraZeneca and Omicron underscored the need for culturally informed public messaging [35]. However, prior research has rarely linked bilingual social media discourse to real-world vaccination uptake at the county level.

This study addresses that gap by analyzing English and Spanish Facebook posts from public pages and groups in Tarrant County, Texas, across 2021 and 2022. Tarrant County is a large, diverse urban county with a substantial Hispanic population (30.5%) [38], making it a useful setting for examining cultural influences on vaccine uptake. By combining bilingual social media data with vaccination data disaggregated by time and ethnicity, this study provides a more granular view of how online discourse relates to population-level vaccination behavior.

The study addresses three research questions:

1. How did stance, misinformation, and topics differ between English and Spanish social media posts regarding COVID-19 vaccines in Tarrant County?
2. How did these differences in stance, misinformation, and topics change over time from 2021 to 2022?
3. What associations existed between the characteristics of these posts and daily vaccination rates among the total and Hispanic populations?

This analysis is informed by two complementary frameworks: (1) the health belief model [39] and (2) the agenda-setting theory [40]. The health belief model suggests that health behaviors are shaped by perceived risk, benefits, barriers, and cues to action [39]. In this context, social media posts may act as cues to action by shaping how people think about vaccine safety, effectiveness, and accessibility. The agenda-setting theory emphasizes the role of media in influencing which issues are seen as important [40]. Applied here, it suggests that frequent discussion of issues, such as side effects, mandates, and misinformation, may increase their salience and thereby shape public attitudes and behaviors. Together, these frameworks provide a useful basis for understanding how vaccine-related discourse on Facebook may be associated with vaccine uptake.

In collaboration with the Tarrant County Public Health Department, this study used monthly vaccination records that included ethnicity, enabling the analysis of both total and Hispanic vaccination uptake. The study also introduced a scalable approach for annotating vaccine-related posts using GPT-4. Post-level variables were defined through iterative manual coding of 200 English and 200 Spanish posts, and these were then used to construct prompts for labeling the full dataset across 16 variables. By examining stance, misinformation, and a wide range of vaccine-related topics, this study has identified message types associated with higher vaccination uptake and highlighted cultural differences that may help guide future public health communication.

### Variables

#### Overview

The analysis included dependent, independent, and control variables. Because Facebook posts may contain more than one relevant characteristic, independent variables were not mutually exclusive.

#### Dependent Variables

*Vaccination* data from January 2021 to December 2022 were obtained from the Tarrant County Public Health Department. The data were anonymized. Each data point represented an individual who got vaccinated and contained information such as the total number of doses received, race, ethnicity, gender, date of birth, and the date of the last dose. It is important to note that these data are not publicly available, and merging these data with social media posts made this research possible. The data were received monthly, where new individuals and doses were added to the record. Such data allowed for the calculation of the total new daily vaccinations at the county level and the new daily vaccinations of the Hispanic population at the county level. New daily vaccinations represent new individuals who have received their first dose that day. Thus, the analysis used new vaccinations per day as a dependent variable. In summary, we defined the dependent variables *new Hispanic vaccinations* and *new total vaccinations* as newly vaccinated Hispanic individuals per day and newly vaccinated individuals in total per day, respectively. Separating the total vaccination rate from the vaccination rate of the Hispanic population was a crucial step in understanding cultural nuances in vaccination uptake, as it allowed the examination of which factors are significant predictors compared with total vaccination uptake. It is important to note that the dependent variables (ie, *new Hispanic vaccinations* and *new total vaccinations*) were calculated by dividing the daily number of first-dose COVID-19 vaccinations by the corresponding population groups in Tarrant County, Texas. Specifically, *new Hispanic vaccinations* were normalized using the county’s Hispanic population, while *new total vaccinations* were normalized using the county’s total population. This standardization (ie, vaccinations per capita) accounts for differences in population size and enables more accurate comparisons over time and between demographic groups.

#### Independent Variables

In the literature, stance is defined as an individual’s standpoint toward a proposition or topic (eg, abortion, feminism, climate change, and others), where the viewpoint can be supporting, opposing, or neutral toward the object [41]. Therefore, the concept of stance could be applied as the users’ standpoint toward vaccination, formulating the first independent variable.

According to multiple research studies, becoming acquainted with antivaccination views and false information on Twitter contributed to vaccine reluctance and denial as well as an overall decrease in the number of individuals receiving the vaccine [42-44]. Thus, *vaccination stance* was used as an independent variable to examine its possible association with the vaccination rate. Encouraging and discouraging posts were included, while neutral posts were not considered. As a neutral stance does not provide any specific insights and does not reflect users’ views toward vaccination, its exclusion allows for a clearer comparison of the associations of encouraging and discouraging posts with the vaccination rate. In more detail, encouraging posts are posts that explicitly support vaccination and aim to motivate others to get vaccinated, while discouraging posts express a negative standpoint toward vaccination and aim to prevent vaccination.

Prior work defined misinformation as unintentionally shared information that is fake or misleading [45]. According to related research, misinformation propagated online hurts COVID-19 vaccine acceptance [46-50]. Some of the most common conspiracy theories regarding COVID-19 vaccines were that they do not work and that they may cause autism or infertility [46]. In addition, with many social media platforms nowadays, there is greater exposure to vaccine misinformation [51], potentially slowing progress toward reaching herd immunity [26]. However, individuals with higher vaccine literacy are less likely to believe in such theories [52,53]. Hence, statistical models used vaccine *misinformation* as an independent variable. Similar to the approach used for stance, only debunking and misinformation posts were included, while posts without relevant content were not considered, as they do not provide useful insights.

Previous research measuring the effectiveness of interventions aiming to reduce vaccine hesitancy toward influenza, human papillomavirus, tetanus, polio, and other vaccines concluded that most interventions included multiple aspects that focus on raising knowledge and vaccine awareness [54]. Many recent studies have proposed the use of social media platforms for targeted messaging to educate the population and enhance awareness in order to reduce vaccine hesitancy [29,46]. Therefore, it is evident that spreading accurate information regarding vaccination can increase vaccination acceptance, and social media could aid in outreach. Thus, it is important to consider which social media posts provide factual information regarding vaccination. Whether a post is *informative* was used as a binary independent variable in the models.

There are several *post topics/categories*. The literature has highlighted multiple reasons behind vaccine hesitancy, such as potential vaccine side effects, mistrust toward the government or health system, doubts about vaccine benefits/efficacy and safety, and dissatisfaction with vaccination mandates [1,5,6,55]. Thus, topics, such as *vaccine side effects*, *government*, *health system*, *vaccine safety*, *policies/mandates*, and *vaccine benefits/efficacy*, were included as independent binary variables in the statistical models. Furthermore, prior work has shown that study participants who knew someone who contracted COVID-19 or died because of it were more likely to receive the vaccine [56]. Therefore, the variable indicating whether a post discusses a COVID-19 illness experience was included as an independent variable. Research has also suggested that religious beliefs play a significant role in vaccine hesitancy, where religiosity is negatively associated with COVID-19 vaccine uptake [57]. Thus, whether a post mentions any religious beliefs was used as a binary independent variable in the models. Finally, vaccine hesitancy is more prevalent across minority populations [14,58]. Therefore, *community-specific advice* variables indicating whether a post includes information specific to any minority population were included as binary variables in the analysis.

While certain variables were identified as independent based on previous literature, others were defined through open-coding techniques during the data labeling process. During the annotation of Facebook posts, annotators identified several frequently occurring categories that were subsequently added as independent variables in the models owing to their potential influence on vaccine uptake. These categories—*vaccine availability*, *education*, *postvaccination advice*, and *statistics*—represent critical aspects of public discourse that address practical, emotional, and informational barriers to vaccination. Vaccine availability posts provide essential details on where, when, and how to get vaccinated, eliminating logistical uncertainties hindering vaccine uptake. Posts related to education discuss school policies and vaccination requirements, which are particularly relevant for parents and students navigating the return to in-person learning. Postvaccination advice posts offer guidance on managing side effects and follow-up doses, addressing anxieties, and simplifying the vaccination process. Finally, posts sharing statistics present concrete data on vaccination rates and COVID-19 cases, serving as powerful motivators by reinforcing the urgency and benefits of vaccination. Including these variables in the models allowed for a more comprehensive analysis of how different types of messaging influence vaccination behavior, providing actionable insights for designing effective, targeted public health interventions.

#### Control Variables

The analysis included a measure of the *population available to vaccinate*, calculated as the share of the relevant population that has not yet received a vaccine. Separate measures were used for the total and Hispanic populations. This variable accounts for the declining pool of eligible first-dose recipients over time.

*Language* was also included as a control variable coded as English or Spanish. This allowed the analysis to test whether post language was associated with differential vaccination uptake while recognizing that language does not map perfectly onto ethnicity.

## Methods

### Overview

**Figure 1.**
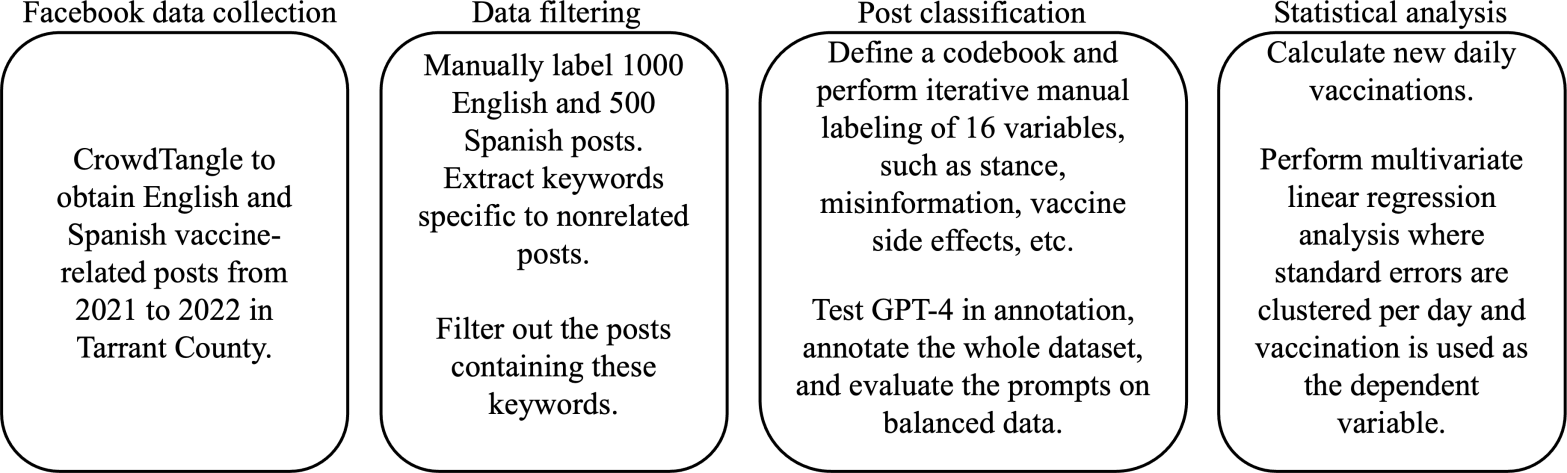
Study framework.

Figure 1 presents the study framework, which included the following 4 stages: Facebook data collection, data filtering, post classification, and statistical analysis.

### Facebook Data Collection

Many studies have leveraged social media platforms to assess vaccine hesitancy and vaccination acceptance. For example, studies have examined sentiments and misinformation about vaccines on platforms like Twitter, Facebook, YouTube, and WhatsApp [29,59-61]. This study collected data from one of the most widely used social media platforms, Facebook [62]. Facebook was considered a suitable platform for this study, as a previous study has reported that 66% of the Hispanic population uses this platform [63]. CrowdTangle application programming interface [64], a social media listening tool from Meta, was leveraged to query the data based on the selected keywords, location (Tarrant County), and period (January 1, 2021, to December 31, 2022). It is important to note that the data provided by CrowdTangle originate from public pages and public groups, not individual users, while the selected location yields posts relevant to that region based on the location distribution of page/group followers or participants. The keywords used for collecting the English dataset were adopted from the CoVaxxy dataset [65], which is an extensive collection of COVID-19 vaccine-related tweets collected with a unique keyword list created by using the snowballing technique. Some examples include *pfizervaccine*, *covax*, and *getvaccinated*. The keywords for collecting the Spanish dataset were adopted from another related study analyzing Spanish Facebook posts in Texas [30], where the keyword list was also created by incorporating a snowballing technique to identify relevant keywords specific to Spanish COVID-19 vaccine-related posts. Some examples include *vacuna covid*, *segunda dosis*, and *efecto secundario covid*. Finally, 21,737 English posts and 1596 Spanish posts were collected. There may be a few reasons for the lower number of Spanish posts. First, the percentage of the Hispanic population in Tarrant County is around 30%. Therefore, the percentage of Spanish versus English posts is expected to be lower. Second, some Hispanic users might still post in English. While this study aimed to compare social media post categories between Hispanic and non-Hispanic populations, a key limitation is the inability to definitively determine the ethnicity of users based on post language. The assumption that Spanish-language posts originate from Hispanic individuals and English-language posts originate from non-Hispanic users may not always be accurate. This introduces potential biases in the analysis, as users may post in a language that does not reflect their ethnicity. This limitation affects the validity of the conclusions drawn about how different demographic groups express attitudes toward vaccination. Despite this, the findings remain relevant because they identify key post categories associated with vaccination uptake in each language group. These insights suggest that the sample is still broadly representative, even if individual user ethnicity cannot be verified with certainty.

### Ethical Considerations

The study was reviewed by the University of Texas at Arlington Institutional Review Board (protocol #2022‐0072), which determined that it did not meet the definition of human subjects research under 45 CFR 46.104 (d)(2i), Revised 2018 [66]. As such, the study was classified as non–human subjects research and did not require Institutional Review Board approval. The project used publicly available data and was consistent with public health surveillance activities, as it involved the systematic collection and analysis of population-level information to monitor health-related behaviors and information patterns, without interaction with individuals or access to identifiable private information, which has been outlined in Centers for Disease Control and Prevention Policy 557 [67]. All Facebook data analyzed in this study were obtained from public Facebook pages and groups only. No private or identifiable user information was collected, and data were analyzed in aggregate and were fully deidentified prior to analysis, ensuring the protection of user privacy and compliance with ethical research standards for the secondary use of data.

### Data Filtering

Although querying based on vaccine-related keywords yielded many Facebook posts, an essential step before further data processing involved ensuring that the posts obtained were related to COVID-19 vaccines. Initially, a random sample of 1000 English posts and 500 Spanish posts was extracted from the whole dataset. Then, 2 pairs of annotators (2 annotators for the English posts and 2 for the Spanish posts) manually labeled the samples by indicating whether each post was related to COVID-19 vaccines. The annotators labeling the English dataset were English speakers, with one annotator having a computer science background and the other having a public health background. The annotators labeling the Spanish dataset were Spanish and English speakers, with one annotator having a computer science background and the other having a public health background. The annotators provided a simple binary label for each post, with “1” used to indicate a relation with COVID-19 vaccines and “0” used to indicate no relation. Once the labeling was completed, the annotators met to discuss and resolve any disagreements in labeling. The Cohen κ scores for the labeling of the English and Spanish datasets were 0.76 (substantial agreement) and 0.14 (slight agreement), respectively. The disagreements in the Spanish sample were manually checked, and the low Cohen κ was found to be mainly due to one of the annotators suggesting that posts of job postings mentioning vaccination requirements in the application process should be considered vaccine-related. However, the other annotators did not agree, as these posts did not provide any further discussion regarding the vaccines. Hence, the annotators decided that these posts should not be classified as vaccine-related. In the English dataset, 60.5% (605/1000) of posts were related to COVID-19 vaccines, while in the Spanish dataset, 83.2% (416/500) of posts were related to the vaccines. By manually examining non–vaccine-related posts, lists of keywords relevant to such posts were created.

Some manually identified keywords specific to non–vaccine-related posts were *dog*, *heartworm*, *hiring*, *music,* etc, corresponding to posts related to animals, events that required COVID-19 vaccination, job openings, or document services. After stemming the texts of the posts and the keywords, posts containing any of the keywords from the corresponding lists were classified as non–vaccine-related. Using this methodology, the percentages of misclassified posts when comparing the obtained labels to the ground-truth dataset were 5.9% (59/1000) and 5.6% (28/500) for the English and Spanish datasets, respectively. Therefore, we filtered the whole dataset using these keywords (ie, discarded posts irrelevant to COVID-19 vaccination). We eventually classified 13,819 English posts and 1249 Spanish posts as relevant.

### Post Classification Ground-Truth Dataset

**Figure 2.**
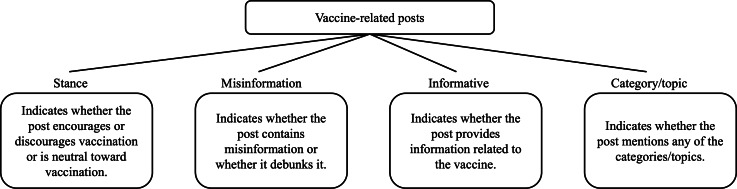
Description of the variables used to annotate the posts.

Previous literature has suggested many reasons for vaccine hesitancy, such as the fear of potential vaccine side effects, efficacy issues, mistrust toward the government or health system, misinformation spread, and others [11,14,26,68]. Thus, this study assessed English and Spanish Facebook posts to investigate how stance, misinformation, informative versus noninformative posts, and post categories/topics (such as vaccine side effects, government, health system, and others) were associated with vaccination rates in Tarrant County in 2021 and 2022. In more detail, the goal was to identify the potential reasons behind vaccine hesitancy in social media posts and examine the association between online activity and actual vaccination uptake. A summary of relevant variables related to social media posts is illustrated in Figure 2.

This study attempted to cover all the reasons for vaccine hesitancy and label them in Facebook posts. However, it is important to note that direct measurement of vaccine trust is challenging, despite its significant role in vaccine reluctance [11]. Therefore, the most prevalent reasons for vaccine hesitancy were incorporated as topics in this study.

Additional classifiers were required to annotate each post with the correct labels for each variable. Initially, random samples of 200 posts were extracted from both the English and Spanish datasets. Then, 2 English speakers labeled the English sample (one with a computer science background and the other with a background in statistics), while 2 Spanish speakers (with public health and computer science backgrounds) and 1 English speaker (with a computer science background), who translated the posts, labeled the Spanish sample using the variables described in Figure 2. The labeling codebook is provided in Multimedia Appendix 1. It was used to train the annotators in performing the labeling. The codebook was initially created by incorporating the knowledge from previous literature, which also helped in defining variables in the Variables section. Manual labeling and defining the codebook were iterative processes. Definitions had to be adjusted multiple times to ensure accuracy and interannotator consistency, and some categories were added by open coding, as discussed in the Independent Variables section. The following are 2 examples from the codebook, including variable definitions and, where applicable, representative examples or supplementary materials:

- Side effects: if the post mentions specific vaccine-related side effects (fever, chills, vomiting, etc; see more here: [69])
- Vaccine availability: if the post mentions anything related to whether vaccinations are available, and where, when, and how. An example post would be “There is a shortage of doses of Pfizer vaccine in Tarrant County.”

If the post was irrelevant to COVID-19 vaccination (as some posts remained after keyword filtering), the annotators did not annotate the post. In some instances, annotators had to open the post. They read the title of the article shared within the post, as sometimes the text of the post did not explicitly mention the vaccine, but it still commented on the article about it. Such posts were considered relevant. After the labeling was completed, the annotators met to resolve any conflicts that were raised in the labeling process to create final labels. However, as 3 annotators labeled the Spanish dataset, the final values were computed by a majority vote.

As the goal of this study was to provide an understanding of the differences in COVID-19 vaccine hesitancy between the English and Spanish datasets, the differences in the observed categories found in these datasets have been further discussed. The percentage of posts in each variable category is reported in Table 1. Moreover, examples of posts in each category can be found in Multimedia Appendix 2.

**Table 1.** Posts containing each of the relevant labels in the manually labeled sample.

Variable	English (n=185), n (%)	Spanish (n=189), n (%)
Stance		
Encouraging	41 (22.2)	14 (7.4)
Discouraging	6 (3.2)	1 (0.5)
Neither	138 (74.6)	174 (92.1)
Category		
Side effects	3 (1.6)	10 (5.3)
Vaccine availability	89 (48.1)	68 (35.9)
Vaccine safety	7 (3.8)	15 (7.9)
Vaccine benefits/efficacy	22 (11.9)	15 (7.9)
COVID-19 illness experience	6 (3.2)	5 (2.6)
Government	32 (17.3)	23 (12.2)
Education	18 (9.7)	5 (2.6)
Health system	52 (28.1)	20 (10.1)
Religion	3 (1.6)	1 (0.5)
Postvaccination advice	4 (2.2)	5 (2.6)
Community-specific advice	8 (4.3)	13 (6.9)
Policies/mandates	50 (27.0)	47 (24.9)
Statistics	23 (12.4)	8 (4.2)
Informative	150 (81.1)	166 (89.2)
Misinformation		
Contains misinformation	0 (0)	0 (0)
Debunks misinformation	4 (2.2)	1 (0.5)

Interestingly, statistics suggested a substantially greater number of posts in the English manually labeled dataset than the Spanish dataset that either encouraged (41/185, 22.2% vs 14/189, 7.4%) or discouraged (6/185, 3.2% vs 1/189, 0.5%) vaccination. This indicates that the majority of posts in the Spanish manually annotated ground-truth dataset were neutral in their stance toward vaccination, which is also implied by the larger percentage of informative posts in this dataset. Furthermore, a larger percentage of Spanish posts discussed vaccine side effects and safety compared with English posts, which tended to discuss matters related to the educational system, government, policy, and health system. This potentially indicates that the Spanish dataset included posts that mainly focused on the vaccine and its relevant information, while the English dataset included many posts relevant to the vaccine but also had posts on other aspects connected to its administration. An additional analysis is required to understand how these relevant categories have been discussed over time and what their associations are with vaccination rates in Tarrant County.

### ChatGPT Labeling

Once the ground-truth dataset was created, the next step involved obtaining labels for the entire dataset. Large language models (LLMs) have been widely used in the natural language processing literature for text classification tasks, while ChatGPT (OpenAI) has been used for data annotation [70-73]. Moreover, GPT-4 has been shown to be a great choice for detecting COVID-19 vaccine self-report and vaccine chatter [74], reaching accuracies of approximately 81% and 93%, respectively, without any additional prompting or fine-tuning. Therefore, it was a suitable candidate for annotating the vaccine-related dataset, considering its powerful ability to understand the context of the provided input and generate correct labels. Thus, the first step involved formulating a prompt that provided a detailed definition of each variable and corresponding examples, which would enable the model to accurately classify each post. Prompt formulation required several iterations, and the initial prompts were modified so that GPT-4 (OpenAI Azure endpoint was used) could better understand the task and generate labels that aligned more with the ground-truth dataset. By adopting a chain-of-thought [75] approach, larger prompts were broken down into multiple smaller, focused prompts, enabling the model to process each variable in steps. Hence, the labeling codebook was used as a starting point, and the same human thought process was followed to create the prompts that could accurately label the data. In more detail, each prompt included the definition of the variable, an example post belonging to the positive class, and a task to classify the post as related to that variable or not, without explanation. In some cases, the prompt had to explicitly tell the model which posts should not fall under the positive class. For example, the following prompt asked GPT-4 to classify a post as policy/mandate-related or not (the post included a definition of the variable, examples of posts that should be labeled as belonging to the positive class, and exceptions, ie, posts that were previously labeled as belonging to the positive class but should belong to the negative class):

*Label the text with “Policy related” or “Not policy related.” “Policy related” should be used if the post mentions vaccination mandates and requirements, authorizations, recommendations, or other COVID-19 policies/mandates issued by health governmental agencies like the CDC or FDA. This includes employee vaccination requirements, vaccine schedule, travel vaccination mandates, mask mandates, vaccine eligibility, vaccine authorizations, social distancing policies, or public health recommendations such as CDC guidelines for vaccination. Posts that primarily mention where vaccines are available for administration or the logistics of vaccine distribution without discussing mandates, requirements, or authorizations should be labeled as “Not policy related.” Respond only with the labels “Policy related” or “Not policy related.” Here is the post:*

This example illustrates the need to provide a very specific prompt to the model to obtain annotations that closely match human labels and do not alternate over multiple iterations. Thus, it was necessary to present the model with a simple task (ie, to provide binary labels) and explain the reasoning aspects that should lead the model to the correct class inference. This sequential breakdown improved accuracy (from 81% to 90% in the case of the policy/mandate variable), as the model could more effectively reason through each part of the task to produce consistent labels. The list of final prompts used to obtain labels for each post in the ground-truth dataset can be found in Multimedia Appendix 3. It is important to acknowledge that this annotation task was not simple even for human annotators, and it required multiple discussions and changes in the codebook. This shows the importance of providing very detailed and precise guidelines to both human annotators and LLMs to obtain highly accurate labels.

Once again, GPT-4 was asked to classify posts as “related” or “not related” to the COVID-19 vaccine as an additional filtering step to remove any noise remaining after the initial keyword filtering. In addition to the post text, the titles of the websites shared in the posts were passed to the prompt along with article/site descriptions to provide additional context that annotators could see during the labeling process. This approach was a more accurate method to detect vaccine-related posts, as in some instances, the post text did not directly mention the COVID-19 vaccine but rather commented on an article about the vaccine. Therefore, such posts were also labeled by annotators as vaccine-related. By providing these inputs, GPT-4 could detect such posts with higher accuracy. The accuracies for detecting vaccine-related and non–vaccine-related posts in the English and Spanish datasets were 99% and 96%, respectively. Before calculating the accuracies for other variables, detected non–vaccine-related posts were removed from the English and Spanish ground-truth datasets, and they remained with 185 and 189 posts, respectively. In addition, some posts that were not accurately detected by the classifier as non–vaccine-related (1 post in the English dataset and 3 posts in the Spanish dataset) were also removed when computing the final model accuracy for the rest of the variables, as such posts were not annotated in the manual labeling process. Moreover, the process of obtaining GPT-4 labels for all variables was repeated thrice to ensure that the labels were consistent, as the “hallucination” of LLMs (generating plausible yet nonfactual content) is a known problem in the literature [76]. The majority vote of 3 labels was used to compute the final labels, as the goal was to check how consistent the output was across multiple iterations. The highest percentages of posts in the English dataset that received different labels in these iterations were found to belong to the vaccine availability (16/200, 8.0%) and government (13/200, 6.5%) categories, while for all the other labels, the value did not exceed 6%, with the lowest being around 1% for COVID-19 illness experience, side effects, and religion. On the other hand, for all the variables in the Spanish dataset, the value did not exceed 6%. When obtaining the labels for the entire datasets, the labels were obtained only once, as a limited number of posts received different labels in multiple iterations.

### Model Evaluation

After the final labels were computed, they were compared with the manually labeled sample. As demonstrated in Table 2, GPT-4 performed very well in labeling the data with the discussed variables. In the English dataset, the highest accuracy of 99% was obtained for detecting vaccine side effect posts, while the lowest accuracy of 89% was obtained for detecting health system–related posts. However, the classification accuracies in the Spanish dataset were slightly different. In some instances, GPT-4 performed better in the Spanish dataset than in the English dataset (eg, education and statistics), but it did not perform as well in detecting vaccine availability. A possible reason for these results is that the Spanish dataset did not contain as many categories as the English dataset. In some cases, this made the categories harder to detect, while in certain instances, the model could easily recognize the absence of categories, yielding a higher accuracy. It is important to acknowledge that most state-of-the-art classifiers for different text classification tasks have certain limitations; hence, it is not surprising that this classifier performs differently in detecting different categories. As the study’s goal did not involve enhancing the state-of-the-art model in detecting the mentioned variables, the classifier’s performance will need to be improved in future work.

**Table 2.** Accuracy of GPT-4 labels for detecting relevant classes in both English and Spanish datasets.

Variable	Accuracy in the English dataset, %	Accuracy in the Spanish dataset, %
Stance	91	89
Category		
Side effects	99	98
Vaccine availability	90	85
Vaccine safety	97	94
Vaccine benefits/efficacy	95	95
COVID-19 illness experience	98	97
Government	93	90
Education	96	98
Health system	89	86
Religion	97	98
Postvaccination advice	97	96
Community-specific advice	95	93
Policies/mandates	90	88
Statistics	91	98
Informative	90	86
Misinformation	93	93

After labels were obtained for the entire dataset, a small number of posts did not have the expected output, and the model could not infer the corresponding label. This issue mainly occurred for very short posts or posts only containing URLs. These posts were discarded from the datasets (62 posts from the English dataset and 23 from the Spanish dataset).

In generating labels for the entire dataset, the English dataset involved 35.2 million input tokens (prompts and posts) and 307,000 output tokens, while the Spanish dataset involved 2.3 million input tokens (prompts and posts) and 37,000 output tokens. The estimated cost of obtaining the annotations for the entire English dataset was US $1074.8, while the cost for the Spanish dataset was US $71.5. The rate limit allowed sending 10 requests per minute; thus, 14,400 prompts could be sent a day. Considering that 11 different prompts were used in this study (with approximately 15,000 posts), approximately 12 days were required to obtain all the annotations. Considering that the posts were collected over a 2-year period, the cost could be considered acceptable in emergency scenarios where a better understanding of reluctance toward interventions could potentially prevent a public health crisis. Moreover, GPT-4 was the latest model released at the time of this study. However, other open-source models could be explored if enough data are available, and this remains a promising area for future work.

### Additional Manual Verification

As noted in Table 1, the distribution of posts for each variable was highly imbalanced. In more detail, most of the time, posts belonging to a negative class for each variable were prevalent in the dataset, which could have led to the misleading accuracies in Table 2. Thus, after completion of labeling of the entire dataset using GPT-4, a random sample of 25 posts belonging to each positive class and 25 posts belonging to each negative class was extracted to verify the accuracy of the annotations. A single annotator labeled 50 posts for each variable (where each class of a multicategorical variable was treated as a binary variable) and compared the labels with the annotations from GPT-4. The results suggested that for most of the variables, GPT-4 annotated 40 or more posts with the accurate label when, in certain cases, GPT-4 labeled all 50 posts correctly (COVID-19 illness experience and religion in the Spanish dataset). In the Spanish dataset, GPT-4 had low accuracy in detecting misinformation (0.70), postvaccination advice (0.60), and discouraging, informative, and community-specific advice (0.76). In the English dataset, the model had high accuracy in detecting religion (0.98), debunking (0.96), statistics (0.96), and vaccine safety (0.94), and had low accuracy in detecting postvaccination advice (0.62), misinformation (0.74), encouraging advice (0.78), and community-specific advice (0.80). For the remaining variables, the model had accuracies higher than 0.80. Therefore, future work should aim to improve the performance of the classifier for these specific annotations. While the use of GPT-4 for annotating the dataset is highly innovative, the lower accuracies observed for some categories suggest room for improvement, which can be addressed in future work. The performances reported in this study are similar to those reported in related work [74], where for detecting COVID-19 vaccine chatter and self-reported vaccination using GPT-4, the accuracies were nearly 93% and 81%, respectively.

Furthermore, another recent study reported that GPT-4 had an average accuracy of 79.2% in various manually labeled datasets [70]. Hence, it is evident that the model performs better for certain variables than others, where fine-tuning might be required. Further labeling of high-quality datasets and ensuring consistency in the labeling process through prompt tuning might be needed to enhance model performance.

### Descriptive Statistics

#### Overview

This section details the number of posts belonging to each category over the time frame of the data collection. The goal was to compare the prevalence of each post category between 2021 and 2022, and between English and Spanish. Finally, combining some of the categories could provide additional insights into the reasons for vaccine hesitancy and how these have changed over time. It is important to note that the posts classified as unrelated by the model were discarded before the analysis. The cleaned English and Spanish datasets consisted of 12,395 and 1123 posts, respectively.

#### English Dataset

The majority of posts in the English dataset were from 2021. In more detail, of the 12,395 posts, 11,088 (89.5%) were shared in 2021 and 1307 (10.5%) were shared in 2022. This decline in 2022 may be attributed to the initial surge in vaccine discussions during the roll-out phase in 2021, when uncertainty and debate were more prominent. By 2022, vaccines were widely available, and public attention may have shifted to other topics. The proportion of posts encouraging vaccination was higher in 2021 than in 2022 (2005/11,088, 18.1% vs 154/1307, 11.8%), while the proportion of discouraging posts was lower in 2021 than in 2022 (624/11,088, 5.6% vs 113/1307, 8.6%). This might indicate that at the beginning of vaccine administration in 2021, there was a greater need to encourage vaccination, and thus, a higher percentage of the population decided to receive the vaccine. On the other hand, in 2022, many people had already received the vaccine, and there was no need for a large encouragement. Similarly, the proportion of informative posts was higher in 2021 than in 2022 (8482/11,088, 76.5% vs 943/1307, 72.1%), which might support the earlier assumption. Finally, the proportion of posts containing misinformation was lower in 2021 than in 2022 (414/11,088, 3.7% vs 79/1307, 6.0%).

Table 3 presents the proportion of encouraging and discouraging posts for each category in 2021 and 2022 in the English dataset. As demonstrated in Table 3, the categories of encouraging and discouraging posts were very different. For instance, the majority of encouraging posts in both 2021 and 2022 were informative, and they mostly discussed vaccine availability and vaccine benefits/efficacy. These posts also promoted the health system, offered advice to specific communities and minorities, and provided useful statistics to encourage vaccination. The topic of education was more common among encouraging posts, which might be due to the high support provided for student vaccination to allow them to continue regular studying activities and in-person classes. On the other hand, discouraging posts in both 2021 and 2022 mostly discussed the government and policies/mandates regarding vaccination, suggesting that the population was hesitant toward the measures taken to mitigate the pandemic, such as vaccination requirements, vaccination passports, and mask mandates. A large proportion of discouraging posts involved misinformation in 2021 (273/624, 43.8%) and 2022 (57/113, 50.4%). A slightly lower proportion of discouraging posts discussed the health system and vaccine safety, and they rarely offered advice or debunked misinformation. Despite these proportions being relatively low, the proportion of posts regarding religion and side effects was higher among discouraging posts than among encouraging posts, suggesting that these factors might still play significant roles in vaccine hesitancy.

The data suggested that misinformation and concerns about government policies were major contributors to vaccine hesitancy, while informative posts and discussions about vaccine availability played critical roles in promoting vaccination.

**Table 3.** Proportion of posts containing each relevant category among encouraging and discouraging posts in 2021 and 2022 in the English dataset.

Variable	2021		2022	
	Encouraging posts (n=2005), n (%)	Discouraging posts (n=624), n (%)	Encouraging posts (n=154), n (%)	Discouraging posts (n=113), n (%)
Side effects	40 (2.0)	36 (5.8)	1 (0.6)	5 (4.4)
Vaccine availability	809 (40.3)	16 (2.6)	77 (50.0)	4 (3.5)
Vaccine safety	130 (6.5)	73 (11.7)	2 (1.3)	13 (11.5)
Vaccine benefits/efficacy	740 (36.9)	26 (4.2)	77 (50.0)	3 (2.7)
COVID-19 illness experience	49 (2.3)	2 (0.3)	1 (0.6)	0 (0)
Government	150 (7.5)	287 (46.0)	14 (9.1)	59 (52.2)
Education	116 (5.8)	25 (4.0)	14 (9.1)	1 (0.8)
Health system	714 (35.6)	132 (21.2)	61 (39.6)	28 (24.8)
Religion	49 (2.4)	32 (5.1)	1 (0.6)	3 (2.7)
Vaccination advice	156 (7.8)	4 (0.6)	10 (6.5)	0 (0)
Community-specific advice	39 (1.9)	10 (1.6)	3 (1.9)	0 (0)
Policies/mandates	361 (18.0)	334 (53.5)	38 (24.7)	70 (61.9)
Statistics	184 (9.2)	18 (2.9)	17 (11.0)	2 (1.8)
Informative	1766 (88.1)	209 (33.5)	148 (96.1)	33 (29.2)
Contains misinformation	10 (0.5)	273 (43.8)	1 (0.6)	57 (50.4)
Debunks misinformation	32 (1.5)	9 (1.4)	1 (0.6)	0 (0)

#### Spanish Dataset

The total number of posts was lower in the Spanish dataset than in the English dataset. Of the 1123 posts, 984 (87.6%) were shared in 2021 and 139 (12.4%) were shared in 2022. The proportion of posts encouraging vaccination was higher in 2021 than in 2022 (158/984, 16.1% vs 14/139, 10.1%), while the proportion of discouraging posts was lower in 2021 than in 2022 (58/984, 5.9% vs 12/139, 8.6%). The proportion of informative posts was higher in 2021 than in 2022 (830/984, 84.3% vs 111/139, 79.9%), while the proportion of posts containing misinformation was lower in 2021 than in 2022 (44/984, 4.5% vs 14/139, 10.1%).

As illustrated in Table 4, encouraging posts in both 2021 and 2022 discussed vaccine availability, benefits/efficacy, the government, and policies, and offered community-specific advice. Interestingly, the topic of the government was more prevalent among encouraging posts than among discouraging posts in both 2021 and 2022, potentially suggesting that mistrust toward the government was not a major cause of vaccine hesitancy in the Hispanic population in Tarrant County. A larger proportion of discouraging posts mentioned policies/mandates compared with encouraging posts. Moreover, a large proportion of discouraging posts discussed vaccine side effects and vaccine safety, revealing additional potential reasons for vaccine hesitancy, which differ from the statistics found in the English dataset, where discouraging posts focused on the government and mandates. Given the smaller dataset, these findings should be interpreted with caution. However, they reveal that vaccine safety and side effects might be significant concerns for the Hispanic population, distinct from the focus on government policies in the English dataset. The health system was a common topic among both encouraging and discouraging Spanish posts; thus, an additional analysis is required to understand the actual relationship between vaccine hesitancy and this topic. Similar to the English dataset, most discouraging posts in the Spanish dataset contained some misinformation. These descriptive insights set the stage for a statistical analysis, which could further explore the relationship between these categories and vaccination rates.

**Table 4.** Proportion of posts containing each relevant category among encouraging and discouraging posts in 2021 and 2022 in the Spanish dataset.

Variable	2021		2022	
	Encouraging posts (n=158), n (%)	Discouraging posts (n=58), n (%)	Encouraging posts (n=14), n (%)	Discouraging posts (n=12), n (%)
Side effects	6 (3.8)	11 (19.0)	0 (0)	2 (16.7)
Vaccine availability	67 (42.4)	1 (1.7)	6 (42.9)	0 (0)
Vaccine safety	8 (5.1)	14 (24.1)	0 (0)	1 (8.3)
Vaccine benefits/efficacy	32 (20.3)	2 (3.4)	3 (21.4)	0 (0)
COVID-19 illness experience	1 (0.6)	2 (3.4)	0 (0)	0 (0)
Government	17 (10.8)	5 (8.6)	2 (14.3)	1 (8.3)
Education	11 (7.0)	1 (1.8)	1 (7.1)	0 (0)
Health system	41 (26.0)	22 (37.9)	5 (35.7)	2 (16.7)
Religion	5 (3.2)	2 (3.4)	0 (0)	0 (0)
Vaccination advice	3 (1.9)	0 (0)	0 (0)	0 (0)
Community-specific advice	16 (10.1)	0 (0)	1 (7.1)	0 (0)
Policies/mandates	23 (14.6)	17 (29.3)	4 (28.6)	4 (33.3)
Statistics	6 (3.8)	4 (6.9)	0 (0)	0 (0)
Informative	143 (90.1)	33 (56.9)	14 (100)	5 (41.7)
Contains misinformation	1 (0.6)	26 (44.8)	0 (0)	8 (66.7)
Debunks misinformation	3 (1.9)	0 (0)	0 (0)	0 (0)

### Statistical Analysis

This study aimed to explore how different characteristics of social media posts are associated with vaccination rates in Tarrant County and compare the trends observed in total vaccination rates and Hispanic vaccination rates. In more detail, the analysis focused on the relationship between different social media post features in English and Spanish and vaccination rates at the daily level throughout 2021 and 2022.

For the analysis, English and Spanish posts were merged into a single dataset (containing a total of 13,518 data points), as there might be Hispanic individuals who post in English rather than Spanish. Merging the English and Spanish datasets required careful attention to both linguistic and cultural nuances in vaccine-related discourse. While the same annotation framework and prompts were applied to both datasets using GPT-4, the way topics are framed can differ significantly across languages and communities. For example, the expression of vaccine skepticism in Spanish posts might often include religious or familial references that could be less common in English posts, requiring more context-aware labeling. Additionally, certain categories, such as government trust or perception of the health care system, may carry different connotations based on cultural background, which could influence how posts are interpreted and categorized. To mitigate these challenges, bilingual annotators reviewed posts and labels in both languages to account for this contextual variation. Nevertheless, we acknowledge that achieving complete semantic and cultural equivalence is challenging, and residual bias in cross-language classification remains a limitation. The dataset was then split into posts originating in 2021 and 2022. For each year, 2 linear regression models corresponding to 2 dependent variables were used by clustering SEs per day, as posts shared on the same day might not be independent of each other. Linear regression was selected for this analysis because the outcome variables (ie, daily total vaccinations and daily Hispanic vaccinations) are continuous, allowing us to estimate the linear relationship between social media content features and vaccination uptake. Since each post is assigned the same vaccination count for its corresponding day, multiple posts share the same value for the dependent variable. This creates “within-day clustering,” where residuals of observations from the same day may be correlated, violating the independence assumption of standard linear regression. To address this, we used “clustered SEs at the day level,” which provide robust inference by correcting for intracluster correlation and ensuring more accurate CIs and *P* values. This approach allows for a valid statistical inference even when the number of posts varies substantially across days.

Furthermore, each data point represented a Facebook post and included the binary categorical variables describing the post. Other variables included in each observation were the date when the post was shared, *new Hispanic vaccinations*, *new total vaccinations* on that particular day, and the language of the post. All 4 models included all the independent and corresponding control variables.

## Results

### Regression Analysis of Social Media Content and Vaccination Outcomes

This section presents the findings of the 4 linear regression models where SEs were clustered per day. It is important to note that similar models were leveraged, but they used the weekly vaccination data rather than the daily data, and SEs were clustered per week. The results of this analysis closely resemble the findings discussed in this section, showing the robustness of the models. Moreover, the models were tested by using a dependent variable, where new vaccinations per day and week were divided by the population available to vaccinate, and the results remained similar. Table 5 presents the significant results of the 4 models in the analysis (variables with nonsignificant findings have been excluded for readability). Complete data are provided in Multimedia Appendix 4.

**Table 5.** Results of the linear regression models.

Variable^a^	Estimate	SE^b^	*P* value
Model 1^c^ (dependent variable: *new total vaccinations* in 2021)			
Vaccine safety - true	4.00 × 10^−4^	1.46 × 10^−4^	.006
Government - true	−1.44 × 10^−4^	6.81 × 10^−5^	.04
Vaccine availability - true	1.58 × 10^−4^	6.19 × 10^−5^	.01
Population available	3.10 × 10^−3^	5.00 × 10^−4^	<.001
Model 2^d^ (dependent variable: *new Hispanic vaccinations* in 2021)			
Encouraging - true	9.88 × 10^−5^	4.25 × 10^−5^	.02
Vaccine safety - true	5.27 × 10^−4^	1.70 × 10^−4^	.002
Government - true	−1.80 × 10^−4^	5.37 × 10^−5^	<.001
Religion - true	2.42 × 10^−4^	8.91 × 10^−5^	.007
Vaccine availability - true	1.01 × 10^−4^	4.89 × 10^−5^	.04
Hispanic population available	1.03 × 10^−3^	4.40 × 10^−4^	.02
Model 3^e^ (dependent variable: *new total vaccinations* in 2022)			
Statistics - true	−5.69 × 10^−5^	1.69 × 10^−5^	<.001
Postvaccination advice - true	7.27 × 10^−5^	3.64 × 10^−5^	.046
Debunking - true	2.01 × 10^−4^	5.36 × 10^−5^	<.001
Population available	1.84 × 10^−2^	1.36 × 10^−3^	<.001
Model 4^f^ (dependent variable: *new Hispanic vaccinations* in 2022)			
Statistics - true	−6.98 × 10^−5^	2.09 × 10^−5^	<.001
Debunking - true	1.94 × 10^−4^	9.56 × 10^−5^	.04
Hispanic population available	1.97 × 10^−2^	1.60 × 10^−3^	<.001

a Models 1 and 2 used data from 2021, while models 3 and 4 used data from 2022.

b SEs were clustered per day.

c *R*2=0.13.

d *R*2=0.04.

e *R*2=0.70.

f *R*2=0.68.

### Trends in 2021

Regression analysis yielded multiple relevant findings. As demonstrated for models 1 and 2 in Table 5, posts discussing vaccine safety were associated with a higher daily increase in total vaccination (*P*=.006) and vaccination of the Hispanic population (*P*=.002). This might indicate that demonstration of vaccine safety once vaccine administration begins is crucial for ensuring a successful intervention. Furthermore, posts mentioning the government were associated with a lower daily increase in both total (*P*=.04) and Hispanic vaccinations (*P*<.001), potentially suggesting mistrust toward the government at that time. In addition, posts providing details about vaccine availability increased the likelihood of a higher number of newly vaccinated individuals (*P*=.01) and Hispanic individuals (*P*=.04). Interestingly, posts about religion showed a statistically significant positive relationship with *new Hispanic vaccinations* (*P*=.007), indicating that such posts might increase the probability of new Hispanic individuals obtaining the vaccine. It is important to note that when running the models where vaccination increase was calculated per week, all variables that showed statistical significance at the daily level remained significant. However, posts regarding the health system showed a negative statistically significant correlation with weekly *new total vaccinations* (*P*=.04) and *new Hispanic vaccinations* (*P*=.03), suggesting potential mistrust toward the health system in this period. Additionally, posts shared in Spanish showed a positive statistically significant correlation with weekly *new Hispanic vaccinations* (*P*=.02), illustrating that posts shared in Spanish were associated with a higher vaccination increase in the Hispanic population compared with posts shared in English.

### Trends in 2022

Models 3 and 4 (Table 5) from 2022 had different results compared with the models from 2021. For example, posts debunking misinformation showed a statistically significant relationship with both *new total vaccinations* (*P*<.001) and *new Hispanic vaccinations* (*P*=.04), demonstrating that such posts are associated with a higher number of new vaccinations compared with nondebunking posts. It is important to note that the difference in findings could be due to the size of the dataset in 2022 compared with the dataset in 2021. In summary, the results (Table 5) indicate that the stance of social media posts, posts debunking misinformation, and some other informational categories, such as vaccine availability and safety, play significant roles in influencing the number of newly vaccinated individuals.

## Discussion

### Principal Findings

This study examined bilingual Facebook discourse about COVID-19 vaccines in Tarrant County and linked it to daily vaccination uptake in the total and Hispanic populations. Three main findings stand out.

First, vaccine-related discourse differed substantially by language and year. English and Spanish posts did not emphasize the same concerns, and the mix of topics shifted from 2021 to 2022. English discouraging posts were dominated by government and policy concerns, whereas Spanish discouraging posts more often emphasized side effects, safety, and the health system. This suggests that vaccine hesitancy was not uniform across language groups and that communication strategies should not assume the same drivers across populations.

Second, some predictors of uptake were common across groups. Posts discussing vaccine availability and vaccine safety were positively associated with vaccination in 2021, and posts debunking misinformation were positively associated with vaccination in 2022. These findings are consistent with the view that practical access information, trust-building content, and correction of false claims are important components of effective vaccine communication.

Third, some predictors were specific to Hispanic vaccination uptake. Encouraging posts and religion-related posts were positively associated with *new Hispanic vaccinations* in 2021, but not with total vaccinations in the same way. This is particularly important because it suggests that culturally grounded content may matter above and beyond general informational messaging.

The significance of religion-related posts is consistent with prior literature showing that religiosity can shape health behavior [57]. In some communities, faith leaders and faith-based institutions serve as highly trusted intermediaries. In such settings, messages framed around protecting family, stewardship, and community responsibility may resonate more strongly than messages framed only in technical or institutional terms. These findings suggest that partnerships with churches and faith-based organizations may be especially valuable in public health outreach to Hispanic communities.

The findings of this study provide several actionable recommendations for public health officials. Social media interventions targeting vaccine-hesitant populations, particularly in the Hispanic community, should be designed with cultural sensitivity in mind [58]. For example, incorporating bilingual content and collaborating with trusted community figures—such as religious leaders or local health care providers—could enhance the credibility and reach of these interventions. Furthermore, posts that emphasize family protection, community well-being, and the dispelling of myths related to vaccine safety and efficacy have been shown to resonate well with Hispanic audiences [77]. By focusing on culturally appropriate messaging and leveraging trusted networks within these communities, public health efforts can more effectively counter vaccine misinformation and build confidence in the vaccination process.

In summary, the results underscore the importance of bilingual communication. Because language may shape how health information is understood and trusted, simply translating English-language messages may not be sufficient. Effective outreach may require culturally aligned framing, not just linguistic conversion.

### Limitations

This study has several limitations. First, the dataset included only posts from public Facebook pages and groups, which may not represent the views of people using private groups, other platforms, or no social media at all. Second, language is not a perfect proxy for ethnicity, and thus, it cannot be assumed that Spanish-language posts come only from Hispanic users or English-language posts come only from non-Hispanic users. Third, because multiple posts shared the same daily vaccination outcome, the observational structure limits causal interpretation even though clustered SEs were used. Fourth, GPT-4 annotation, while strong overall, was not perfect and may have introduced measurement errors in some categories. Finally, the models did not fully capture broader cultural, structural, or offline influences on vaccine behavior.

### Conclusions

This study examined how stance, misinformation, and topic content in English and Spanish Facebook posts were associated with new daily vaccinations in Tarrant County, Texas, during 2021 and 2022. The findings showed that social media discourse differed meaningfully across languages and over time and that some message types were associated with higher vaccination uptake. Across both populations, posts addressing vaccine availability, safety, and misinformation debunking were associated with higher uptake. For the Hispanic population specifically, encouraging posts and religion-related posts were also significant predictors, suggesting culturally specific dynamics.

Taken together, these findings support the use of targeted, culturally responsive, and bilingual public health messaging in future emergencies, and they suggest that social media analysis can help identify which kinds of messages may be most useful for reaching specific communities.

## Supplementary material

10.2196/72465 Multimedia Appendix 1 Labeling guidelines.

10.2196/72465 Multimedia Appendix 2 Example posts for each category.

10.2196/72465 Multimedia Appendix 3 List of prompts.

10.2196/72465 Multimedia Appendix 4 Full regression tables.

## Abbreviations

LLM: large language model
WHO: World Health Organization
