# Supplementary material for: Assessment of the Cultural Nuances in COVID-19 Vaccine Uptake Through a Comparative Analysis of English and Spanish Facebook Posts in Tarrant County, Texas: Longitudinal Study
Source: Online J Public Health Inform. 2026 Apr 27;18:e72465. doi: 10.2196/72465 (PMC13117226; doi:10.2196/72465)
Supplement: Multimedia Appendix 1 [file ojphi-v18-e72465-s001.docx]

## Multimedia Appendix 1

Below are the definitions and possible labels that belong to each variable which were also provided to each annotator as labeling guidelines:

- Stance – a multi-class variable that indicates whether the post encourages the vaccine or not. The post can have one of the following labels:
  - Encouraging – if the post encourages others to get vaccinated (claims positive things about the vaccine). An example would be “I received my first dose yesterday. I feel amazing!”
  - Discouraging – if the post discourages others from getting vaccinated (claims negative things about the vaccine). An example would be “I do not trust our health system. The vaccines were developed way too fast.”
  - Neither – the post does not encourage or discourage vaccination (examples are purely informative posts, such as “1.3 million people are vaccinated now”).
- Post Category – a multi-class variable that describes what the post is focusing on. One post can contain multiple categories. The categories are the following:
  - Side effects – if the post mentions specific vaccine-related side effects (fever, chills, vomiting, etc. See more here: https://www.cdc.gov/vaccines/hcp/vis/vis-statements/COVID-19.html).
  - Vaccine availability - if the post mentions anything related to whether vaccinations are available or not, and where/when/how. An example post would be “There is a shortage of doses of Pfizer vaccine in Tarrant County”.
  - Vaccine safety – if the post talks about the safety of the vaccine (becoming sterile, tracking, cardiovascular heart attacks, rapid vaccine development, lack of vaccine testing, etc.). For more details about vaccine safety, click here: https://www.who.int/news-room/feature-stories/detail/safety-of-covid-19-vaccines.
  - Vaccine benefits/efficacy – if the post discusses the benefits of receiving a vaccine. For example “getting the vaccine will significantly reduce the impact of COVID-19 symptoms”.
  - COVID-19 illness experience – if the post shares the experience of the person when suffering from COVID-19. For example, “I received a positive test and I felt terrible – my whole body was in pain and I could not take a deep breath”.
  - Government – if the focus of the post is political/governmental discussion. An example post would be “The U.S. government is considering giving some people half the dose of Moderna’s COVID-19 vaccine in order to speed vaccinations” or “President-elect Joe Biden will release most available COVID-19 vaccine doses to speed delivery to more people, a reversal of the Trump administration policy, his office said Friday.” This does not include health-related governmental agencies, these should belong to the Health System.
  - Education – if the focus of the post is a discussion related to the education system. An example would be “Students need to be vaccinated to be able to go back to school”.
  - Health system - if the focus of the post is a discussion related to the health system. An example would be “I cannot get vaccinated as I do not have insurance and there is a shortage of nurses”, as well as posts mentioning recommendations from CDC or FDA.
  - Religion – if the post mentioned the reasons for or against vaccination related to religion. An example would be “I do not need to get vaccinated as God protects me from the virus”.
  - Vaccination advice – if the post offers health and wellness vaccination advice, such as “Wearing a mask after vaccination is still mandatory”. For examples, click here: https://www.cdc.gov/vaccines/videos/coronavirus/expect-after-getting-COVID-19-vaccine-transcript.pdf.
  - Community Specific Advice - if the post contains targeted information addressing concerns and needs of a specific population/demographic group (Hispanic, Black, etc.). An example would be “There will be a specific vaccination site for a population that speaks Spanish only, so everyone can get accurate information regarding COVID-19 vaccines”.
  - Policy – if the post talks about current vaccination mandates and requirements. An example would be “All the employees have to be vaccinated against COVID-19” or “All passengers need to be vaccinated to enter the country”.
  - Statistics - if the post shares any statistics related to the number of COVID-19 cases, number of vaccinations, number of deaths, etc.
- Informative – a binary variable that indicates whether the post contains information related to vaccination. An example would be “All the employees have to be vaccinated against COVID-19”.
- Misinformation – a multi-class variable indicating whether there is some misinformation in the post or not. The post can have one of the following labels:
  - Misinformation – if the post contains misinformation. An example would be “I do not want to become infertile, therefore, I will not get vaccinated”.
  - No misinformation – the post does not contain misinformation. An example would be “I received my first dose yesterday and I have a fever. However, that is a common side effect”.
  - Debunking – if the post tries to debunk the misinformation in some way. An example would be “Everyone who thinks the Government is putting microchips in them through vaccines is crazy. Go get the vaccine!”
