# Supplementary material for: Assessment of the Cultural Nuances in COVID-19 Vaccine Uptake Through a Comparative Analysis of English and Spanish Facebook Posts in Tarrant County, Texas: Longitudinal Study
Source: Online J Public Health Inform. 2026 Apr 27;18:e72465. doi: 10.2196/72465 (PMC13117226; doi:10.2196/72465)
Supplement: Multimedia Appendix 2 [file ojphi-v18-e72465-s002.docx]

# Multimedia Appendix 2

Examples for each variable in the dataset.

| Category | Example post |
| --- | --- |
| Stance (encouraging) | “Please Share—Vaccinate your kids (12+), yourself, your neighbors all at the same time.  Let’s enjoy our summer, keep each other safe and healthy - and put this pandemic in the past.  Appts. or walk-in at your convenience at John Clark Stadium tomorrow, Thursday and Friday.  The shots are safe and work! Got mine and so did my 13-year old - woohoo! 😊” |
| Stance (discouraging) | “While millions of people attend sporting events across the country, we are still seeing the radicals push for job-killing vaccine mandates. Americans are over COVID…it’s time for the tyranny to stop.” |
| Category (side effects) | “So far, experts say about 30% of those who get one of three COVID-19 vaccines experience mild to moderate symptoms, but those are actually a sign the vaccine is working.” |
| Category (vaccine availability) | “All 3 COVID vaccine's will be available at the E. Arlington Public Library & Rec Center - 1817 New York Ave on Friday from 9 to 4. ArlingtonTx Fire will be administering doses, no appointment necessary. https://arlingtontx.gov/.../coronavirus_vaccine_clinic...” |
| Category (vaccine safety) | “😂🤣😂🤣😂China is spreading conspiracy theories that the coronavirus was created in an American lab and that a US-created vaccine is killing the elderly” |
| Category (vaccine benefits/efficacy) | “Pfizer’s COVID-19 vaccine for children ages 5 to 11 years old is now available in Tarrant County. Parents and legal guardians should first talk to their pediatrician or local pharmacy to obtain their vaccination. Tarrant County Public Health will also be offering the children’s vaccine for those who cannot obtain it elsewhere.  The Pfizer COVID-19 vaccine received final approval for use with children from the CDC on Tuesday, Nov. 2, 2021. On Oct. 29 the vaccine received its Emergency Use Authorization from the FDA. The FDA and CDC reviewed extensive data from trials to ensure the vaccine is safe for younger children. Critical points found during the studies include:  +Effectiveness: Immune responses of children 5 - 11 years of age were comparable to those of individuals 16 through 25. In addition, the vaccine was found to be 90.7 percent effective in preventing COVID-19 in children 5 - 11.  +Safety: The vaccine’s safety was studied in approximately 3,100 children age 5 - 11 who received the vaccine and no serious side effects have been detected in the ongoing study.  +Dosage: The dose is just one-third (10 micrograms) of the dose for those 12 years of age and older (30 micrograms).  Children age 5 – 11 will receive two doses, 21 days apart, similar to those in the older groups already vaccinate. If a child cannot receive their second dose at 21 days, they are encouraged to get their second dose as soon as possible. Once the series has begun, there is no reason to restart; just finish up when it is possible. At this time, a booster dose is not recommended for children.” |
| Category (COVID-19 illness experience) | “So if you are still hesitant to get the vaccine, I have a story for you about my parents...  Two weeks ago my mom, who is double-vaxxed and boosted, thought she only had a cold for a few days and didn't bother to get tested or quarantine herself. A few days later my dad, who is not vaccinated, start having symptoms but only A LOT worse and took an at-home test that showed he was positive. Turns out that mom unknowingly had covid and my dad caught it too. The only place my mom thinks she caught it from was the grocery as she hadn't been around anyone or left the house for almost a week (other than the grocery) before her symptoms started.  Of course dad is a stubborn one and refused to go to the hospital, despite clearly being very ill for several days, until this morning when my mom called 911 because he wasn't able to form full sentences and had a low O2 level, and has now been admitted with pneumonia from the virus. If he had been vaccinated he would have most likely had a similar experience to my mother and avoided being in the hospital.  GET VACCINATED!!!!!  Also, according to a billboard a friend once saw, "The leading killer of men is refusing to see a doctor." This needs to stop.” |
| Category (Government) | “The Supreme Court has stopped the Biden administration from enforcing a requirement that employees at large businesses be vaccinated against COVID-19 or undergo weekly testing and wear a mask on the job.  At the same time, the court is allowing the administration to proceed with a vaccine mandate for most health care workers in the U.S. https://www.wfaa.com/.../507-184eed7d-af4b-4c96-bcd7...” |
| Category (education) | “Vaccines remain our best defense against COVID‑19.  Students who come get their vaccine are eligible for incentives as part of the Mav Motivator Program.  The next clinic is open tomorrow (Sep. 30) from 9am to 12pm in the MAC  Learn more HERE ▶️ https://bit.ly/37UEd6c” |
| Category (health system) | “Trinity Metro and Tarrant County are teaming up to provide free rides to customers who are traveling to COVID-19 vaccination sites.  To obtain a free ride, passengers will need to show the operator or train conductor the appointment text or email from Tarrant County Public Health.  https://ridetrinitymetro.org/free-rides-to-covid-19.../...” |
| Category (religion) | “Organizers were giving vaccines out to congregation members, as reports show that at least 40% of evangelical Christians are wary about getting a shot.” |
| Category (post-vaccination advice) | “Taking certain medications before getting the COVID-19 vaccine can lessen your immune response to it.” |
| Category (Community Specific Advice) | “Our colleagues at Tarrant County Public Health created this video in Spanish to help encourage everyone in our community to get the COVID vaccine when it is their turn.  Register on the TCPH website: https://www.tarrantcounty.com/.../COVID-19-Vaccine.html” |
| Category (Policy/mandates) | “The Texas establishment does not seem to understand what the governor apparently now understands. There is sufficient numbers of Texas workers who are willing to draw the line in the sand on employer vax mandates that serious damage to the economy of Texas, the lives of Texans, and the fortunes of Texans if we do not figure out how to stop the federal vax mandates.” |
| Category (Statistics) | "(CNN) On Thursday, President Joe Biden announced that the United States will donate 500 million doses of the Pfizer Covid-19 vaccine worldwide, dramatically building on a previous commitment to share 80 million vaccines." |
| Informative | “Do you have questions about the COVID vaccines and pregnancy? One physician discusses why she recommends getting vaccinated if you are pregnant:” |
| Misinformation (contains misinformation) | “Samantha Wendell was worried the vaccine would affect her fertility. Instead of attending her wedding, her family is now planning her funeral.” |
| Misinformation (debunks misinformation) | “With so much information about COVID-19 vaccination, you might be overwhelmed. During April’s COVID-19 Vaccine Awareness & Education Month, we are providing some information that’s backed by the medical community and the CDC.  Myth: A COVID-19 vaccine can make me sick with COVID-19.  Truth: According to the Centers for Disease Control and Prevention, none of the authorized and recommended COVID-19 vaccines or COVID-19 vaccines currently in development in the U.S. contain the live virus that causes COVID-19. This means that a COVID-19 vaccine cannot make you sick with COVID-19.  There are several different types of vaccines in development. All of them teach our immune systems how to recognize and fight the virus that causes COVID-19. Sometimes this process can cause symptoms, like fever. These symptoms are normal and are a sign that the body is building protection against the virus that causes COVID-19.” |
