# Supplementary material for: Assessment of the Cultural Nuances in COVID-19 Vaccine Uptake Through a Comparative Analysis of English and Spanish Facebook Posts in Tarrant County, Texas: Longitudinal Study
Source: Online J Public Health Inform. 2026 Apr 27;18:e72465. doi: 10.2196/72465 (PMC13117226; doi:10.2196/72465)
Supplement: Multimedia Appendix 3 [file ojphi-v18-e72465-s003.docx]

## Multimedia Appendix 3

Below are prompts passed to GPT-4 to obtain the labels for the English and Spanish datasets. Note that the third prompt obtains Community-specific advice labels. However, the accuracy of the obtained labels was further improved by running the additional prompt. Therefore, the third prompt was not modified as the accuracy of the other two variables in the prompt (COVID-19 illness experience and Post-Vaccination Advice) were above 90%.

- 'Based on the text of this post, assign one or more of the topics from the list that are relevant to the post but they must be directly mentioned. Here are the definitions of each topic that you should closely consider when labeling each post: Vaccine safety: If the post talks about the safety of the vaccine, including concerns about potential adverse effects or regulatory actions. Example: "The FDA and CDC called for a pause on the Johnson & Johnson COVID-19 vaccine due to safety concerns". Vaccine side effects: If the post mentions specific vaccine-related side effects that recipients may experience. Example: "Some recipients of the Moderna vaccine reported mild side effects such as fever and fatigue". Vaccine benefits/efficacy: If the post discusses the benefits of receiving a vaccine. Example: "Getting the Pfizer vaccine significantly reduces the risk of severe COVID-19 symptoms". Respond by providing a list of topics separated by a comma, no explanation is needed. In case none of the topics apply, return None. Here is the text of the post:'
- 'Based on the text of this post, assign one or more of the topics from the list that are relevant to the post but they must be directly mentioned. Here are the definitions of each topic that you should closely consider when labeling each post: Government should be used if the focus of the post is political/governmental discussion, such as mentioning political figures, political parties, and others. However, governmental health agencies should not fall into this category. An example post related to the Government would be:"The U.S. government is considering giving some people half the dose of Modernas COVID-19 vaccine in order to speed vaccinations" or "President-elect Joe Biden will release most available COVID-19 vaccine doses to speed delivery to more people, a reversal of the Trump administration policy, his office said Friday". Topic Education should be used if the focus of the post is a discussion related to the education system. An example would be "All students can go back to school after getting vaccinated". Religion should be used the post mentioned the reasons for or against vaccination related to religion. An example would be "I do not need to get vaccinated as God protects me from the virus". Respond with the list of labels separated by a comma. No explanation is needed. In case none of the topics apply, return None. Here is the text of the post:'
- 'Based on the text of this post, assign one or more of the topics from the list that are relevant to the post but they must be directly mentioned. Here are the definitions of each topic that you should closely consider when labeling each post: COVID-19 illness experience should be used only if the post shares the experience of the person when suffering from COVID-19. For example, "I received a positive test and I felt terrible as my whole body was in pain and I could not take a deep breath", however, it should not include a report on the number of COVID-19 cases. Vaccination advice should be used if the post offers health and wellness post-vaccination advice, such as "After receiving a vaccine, drink plenty of fluids and you can resume activities without wearing a mask". Community Specific Advice should be used if the post contains targeted information addressing concerns and needs of minority populations, such as Hispanic, Black, Indigenous, or other specific minority groups. An example would be that there will be a specific vaccination site for the Hispanic population, providing bilingual information regarding COVID-19 vaccine. Statistics should be used if the post shares any statistics related to the number of COVID-19 cases, number of vaccinations, number of deaths, etc. Example: "Millions of Americans received a first shot of the vaccine". Respond with the labels separated by a comma. No explanation is needed. In case none of the topics apply, return None. Here is the text of the post:'
- 'Based on the text of this post, label a variable named Misinformation with one of the following labels: Misinformation if the post contains misinformation. An example would be "I do not want to become infertile, therefore, I will not get vaccinated". No misinformation if the post does not contain misinformation. An example would be "I received my first dose yesterday and I have a fever. However, that is a common side effect". Debunking if the post tries to debunk the misinformation in some way. An example would be "Everyone who thinks the government is putting microchips in them through vaccines is crazy. Go get the vaccine!" Respond only with the label. No explanation is needed. Here is the text of the post:'
- 'Label the provided text with one of the following labels according to the expressed standpoint toward vaccination: Encouraging if the post encourages others to get vaccinated (e.g., claims positive things about the vaccine and calls for action). Example: "I received my first dose yesterday and I feel amazing! You should do it too!" or "CDC recommends getting vaccinated if you are pregnant!". Additionally, posts that highlight the positive impact of vaccines or show prominent figures getting vaccinated, thereby potentially motivating others, should also be labeled as encouraging. Discouraging if the post discourages others from getting vaccinated (e.g., claims negative things about the vaccine that might discourage others from getting a vaccine). Example: "I do not trust our health system. The vaccines were developed way too fast. I will not receive the vaccine" or "Vaccine mandate is tyranny against humanity. Why are vaccines mandatory?". Neither if the post neither encourage nor discourages vaccination (e.g., purely informative posts that do not directly suggest others to get or not get vaccinated). Example: "1.3 million people are vaccinated now" or "There is a new vaccination site open" or "President said there will be more doses available soon". Respond only with the appropriate label. No explanation is needed. Here is the text of the post: '
- 'Label the text with "Informative" or "Not Informative". "Informative" should be used if the post contains news or information related to vaccination, including but not limited to vaccine mandates, eligibility, vaccination sites, as well as other news articles or statements that provide factual information about COVID-19. Respond only with the labels "Informative" or "Not Informative". Here is the post:'
- 'Label the text with "Policy related" or "Not policy related". "Policy related" should be used if the post mentions vaccination mandates and requirements, authorizations, recommendations, or other COVID-19 policies/mandates issued by health governmental agencies like the CDC or FDA. This includes employee vaccination requirements, vaccine schedule, travel vaccination mandates, mask mandates, vaccine eligibility, vaccine authorizations, social distancing policies, or public health recommendations such as CDC guidelines for vaccination. Posts that primarily mention where vaccines are available for administration or the logistics of vaccine distribution without discussing mandates, requirements, or authorizations should be labeled as "Not policy related". Respond only with the labels "Policy related" or "Not policy related". Here is the post:'
- 'Label the text with "Health system related" or "Not Health system related". "Health system related" should be used if the post mentions directives from health agencies like the CDC and FDA or posts mentioning healthcare professionals (doctors, nurses) and public health departments. The post is not "Health system related" if it just mentions vaccine schedule, vaccine availability at a certain health system unit, vaccine eligibility, or other COVID-19-related policy changes. Respond only with "Health system related" or "Not Health system related". Here is the post: '
- 'Label the text with “Vaccine availability related” or “Not vaccine availability related”. “Vaccine availability related” should be used if there is a direct mention of vaccine availability and accessibility, specific locations, groups for availability, new vaccinations, vaccine registration websites, or eligibility; implied availability or focus on prioritization should be labeled as “Not vaccine availability related”. Examples of "Vaccine availability related" include: “There is a shortage of doses of Pfizer vaccine in Tarrant County” and “The FDA and CDC called for a pause on the Johnson & Johnson COVID-19 vaccine due to safety concerns”. Respond with the labels only. No explanation is needed. Here is the post:'
- 'Based on the text of this post, label a post as "Not Related" in case the post does not mention or is not related directly to the COVID-19 vaccine, or if it only mentions a COVID-19 vaccine requirement for a job without discussing the vaccine itself. Posts that mention pro-vaccine or anti-vaccine information should be labeled as "Related." Respond only with the labels "Related" or "Not Related" only. Here is the text of the post:'
- 'Label the post as "yes" only if it explicitly mentions or addresses the concerns of specific minority populations, such as Hispanic, Black, Indigenous, or other ethnic minority groups. If the post discusses geographical locations or general events without directly referencing the specific needs of these groups, label it as "no". General mentions of locations, even if they involve minority regions, do not qualify unless they explicitly address the concerns of the minority populations. Avoid interpreting general health or job opportunities as related to minority groups unless they are clearly referenced. Respond only with yes or no. No explanation is needed. Here is the text of the post:'
