# Supplementary material for: Assessment of the Cultural Nuances in COVID-19 Vaccine Uptake Through a Comparative Analysis of English and Spanish Facebook Posts in Tarrant County, Texas: Longitudinal Study
Source: Online J Public Health Inform. 2026 Apr 27;18:e72465. doi: 10.2196/72465 (PMC13117226; doi:10.2196/72465)
Supplement: Multimedia Appendix 4 [file ojphi-v18-e72465-s004.docx]

## Multimedia Appendix 4

Model 1. Relationship between post-related variables and *new-Total-vaccinations* in 2021.

| Term | Estimate | Std. Error | t-value | P - value |
| --- | --- | --- | --- | --- |
| Intercept | -0.0004 | 0.0003 | -1.511 | .131 |
| Encouraging true | 5.2e-05 | 4.8e-05 | 1.089 | .276 |
| Discouraging true | -7.5e-05 | 5.9e-05 | -1.281 | .2 |
| Vaccine benefits/efficacy true | -2.5e-05 | 6.5e-05 | -0.391 | .696 |
| Vaccine safety true | 0.0004 | 0.0001 | 2.74 | .006 |
| Vaccine side effects true | -8.5e-05 | 0.0001 | -0.696 | .486 |
| Education true | -0.0001 | 0.0001 | -1.107 | .268 |
| Government true | -0.0001 | 6.8e-05 | -2.108 | .035 |
| Religion true | 0.0002 | 0.0001 | 1.848 | .065 |
| Community-specifica dvice true | 2.5e-05 | 0.0001 | 0.248 | .804 |
| Statistics true | -3e-06 | 5.6e-05 | -0.053 | .958 |
| Post-vaccination advice true | 5.5e-05 | 8.7e-05 | 0.635 | .526 |
| Misinformation true | -1.9e-05 | 6.7e-05 | -0.275 | .783 |
| Debunking true | -0.0002 | 0.0001 | -1.614 | .106 |
| Informative true | 3e-06 | 3.6e-05 | 0.081 | .936 |
| Policy true | 6.9e-05 | 6.7e-05 | 1.023 | .306 |
| Health system true | -8.6e-05 | 5.2e-05 | -1.64 | .101 |
| Vaccine availability true | 0.0002 | 6.2e-05 | 2.548 | .010 |
| Language Spanish | 2.2e-05 | 4.8e-05 | 0.453 | .651 |
| Total Population Available | 0.003 | 0.0005 | 6.192 | P < .001 |

Model 2. Relationship between post-related variables and *new-Hispanic-vaccinations* in 2021.

| Term | Estimate | Std. Error | t-value | P - value |
| --- | --- | --- | --- | --- |
| Intercept | 0.0007 | 0.0003 | 2.208 | .027 |
| Encouraging true | 9.9e-05 | 4.3e-05 | 2.323 | .02 |
| Discouraging true | -5.9e-05 | 5e-05 | -1.182 | .237 |
| Vaccine benefits/efficacy true | -2.6e-05 | 5.9e-05 | -0.437 | .662 |
| Vaccine safety true | 5.3e-04 | 1.7e-04 | 3.117 | .002 |
| Vaccine side effects true | -1.2e-05 | 0.0001 | -0.108 | .914 |
| Education true | 3.3e-05 | 8.7e-05 | 0.38 | .704 |
| Government true | -1.8e-04 | 5.4e-05 | -3.344 | P < .001 |
| Religion true | 2.4e-04 | 8.9e-05 | 2.711 | .007 |
| Community-specific advice true | -4e-06 | 9e-05 | -0.049 | .961 |
| Statistics true | 2.1e-05 | 4.5e-05 | 0.469 | .639 |
| Post-vaccination advice true | -1.2e-05 | 7.4e-05 | -0.168 | .866 |
| Misinformation true | -4.7e-05 | 5.8e-05 | -0.803 | .422 |
| Debunking true | -1.9e-04 | 0.0001 | -1.654 | .098 |
| Informative true | -4.9e-05 | 3.2e-05 | -1.559 | .119 |
| Policy true | 7e-05 | 5.6e-05 | 1.234 | .217 |
| Health system true | -8e-05 | 4.3e-05 | -1.878 | .06 |
| Vaccine availability true | 0.0001 | 4.9e-05 | 2.07 | .038 |
| Language Spanish | 6.3e-05 | 4.3e-05 | 1.46 | .144 |
| Hispanic population available | 0.001 | 0.0004 | 2.336 | .019 |

Model 3. Relationship between post-related variables and *new-Total-vaccinations* in 2022.

| Term | Estimate | Std.Error | t-value | P - value |
| --- | --- | --- | --- | --- |
| intercept | -0.007 | 0.0006 | -13.109 | P < .001 |
| Encouraging true | 1.9e-05 | 1.9e-05 | 1.004 | .315 |
| Discouraging true | 1.2e-05 | 3e-05 | 0.391 | .696 |
| Vaccine benefits/efficacy true | -1.9e-05 | 1.9e-05 | -1.019 | .308 |
| Vaccine safety true | -3.4e-05 | 3.7e-05 | -0.916 | .36 |
| Vaccine side effects true | 4.5e-05 | 5e-05 | 0.909 | .364 |
| Education true | -3.4e-05 | 2.8e-05 | -1.211 | .226 |
| Government true | 1.1e-05 | 2.3e-05 | 0.487 | .626 |
| Religion true | 4e-06 | 4e-05 | 0.109 | .913 |
| Community-specific advice true | -1e-06 | 3.7e-05 | -0.021 | .983 |
| Statistics true | -5.7e-05 | 1.7e-05 | -3.358 | P < .001 |
| Post-vaccination advice true | 7.3e-05 | 3.6e-05 | 1.997 | .046 |
| Misinformation true | -2.7e-05 | 2.7e-05 | -0.977 | .329 |
| Debunking true | 2e-04 | 5.4e-05 | 3.761 | P < .001 |
| Informative true | -9e-06 | 1.5e-05 | -0.617 | .537 |
| Policy true | 8e-06 | 1.8e-05 | 0.434 | .664 |
| Health system true | -5e-06 | 1.3e-05 | -0.344 | .731 |
| Vaccine availability true | 2e-06 | 1.4e-05 | 0.156 | .876 |
| Language Spanish | -3.6e-05 | 2.1e-05 | -1.713 | .087 |
| Total population available | 0.018 | 0.001 | 13.494 | P < .001 |

Model 4. Relationship between post-related variables and *new-Hispanic-vaccinations* in 2022.

| Term | Estimate | Std.Error | t-value | P - value |
| --- | --- | --- | --- | --- |
| intercept | -0.009 | 0.0008 | -11.987 | P < .001 |
| Encouraging true | 1.5e-05 | 2.2e-05 | 0.679 | .497 |
| Discouraging true | 2.8e-05 | 3.6e-05 | 0.793 | .428 |
| Vaccine benefits/efficacy true | -1.8e-05 | 2.2e-05 | -0.801 | .423 |
| Vaccine safety true | -2.1e-05 | 4.6e-05 | -0.446 | .655 |
| Vaccine side effects true | 7.6e-05 | 6.2e-05 | 1.218 | .223 |
| Education true | -4.9e-05 | 3.2e-05 | -1.531 | .126 |
| Government true | 1.4e-05 | 2.5e-05 | 0.572 | .567 |
| Religion true | -2.7e-05 | 4.5e-05 | -0.592 | .554 |
| Community-specific advice true | -6e-06 | 3.9e-05 | -0.155 | .877 |
| Statistics true | -7e-05 | 2.1e-05 | -3.337 | P < .001 |
| Post-vaccination advice true | 4.8e-05 | 3.9e-05 | 1.221 | .222 |
| Misinformation true | -3.1e-05 | 3.1e-05 | -1.001 | .317 |
| Debunking true | 0.0002 | 9.6e-05 | 2.033 | .042 |
| Informative true | -1.4e-05 | 1.7e-05 | -0.812 | .417 |
| Policy true | -6e-06 | 2.1e-05 | -0.272 | .785 |
| Health system true | 1.3e-07 | 1.7e-05 | 0.007 | .994 |
| Vaccine availability true | -4e-06 | 1.6e-05 | -0.251 | .801 |
| Language Spanish | -2.1e-05 | 2.6e-05 | -0.822 | .411 |
| Hispanic population available | 0.02 | 0.002 | 12.245 | P < .001 |
